# Leveraging microbiota-metabolites to reduce inflammation and promote functional recovery following spinal cord injury in female mice

**DOI:** 10.1016/j.bbih.2025.101157

**Published:** 2025-12-02

**Authors:** Ashley J. Douthitt, Aaron Bennett, Alexandra Koustova, Asim Abdelfattah, Darijana Horvat, Cédric G. Geoffroy

**Affiliations:** a Department of Neuroscience and Experimental Therapeutics, Texas A&M University Health Science Center, Bryan, TX, 77807, United States; b Wolfson Sensory, Pain and Regeneration Centre (SPaRC), King's College London, London, SE1 1UL, United Kingdom

**Keywords:** Spinal cord injury, Tryptophan, Indole, Inflammation, Recovery, Locomotion, Liver, Metabolite

## Abstract

Spinal cord injury induces extensive neurological impairment and drives systemic and tissue-level inflammatory responses that accelerate secondary systemic damage. Emerging evidence suggests that gut microbiota-derived metabolites can influence post-injury inflammation, presenting a potential therapeutic approach. This study examines whether the tryptophan-derived metabolites indole and indole-3-propionic acid modulate inflammatory responses and improve outcomes following spinal cord injury. Female C57BL/6J mice received a severe thoracic-8 contusion-compression injury and were administered indole or indole-3-proprionic acid daily via oral gavage for the duration of the observation period. In an acute cohort, 7 days post-injury, neither treatment altered plasma inflammatory profiles relative to injury controls. However, both metabolites significantly reduced CD68^+^ macrophage presence within the injured spinal cord. In a chronic cohort, 42 days post-injury, metabolite treatment mitigated injury-induced body composition changes, improved locomotor recovery and reduced inflammatory pathologies within the liver and spinal cord. These findings identify gut-derived metabolites as a promising therapeutic strategy targeting the gut-spinal cord axis to attenuate systemic injury mechanisms and support recovery.

## Highlights

• Microbial metabolites IPA and indole reduce liver and spinal inflammation post-SCI.
• IPA preserved metabolic balance after SCI.
• IPA improved locomotor recovery after SCI.
• Microbial metabolites show therapeutic potential for SCI.

## 1 Introduction

Spinal cord injury (SCI) leads to profound disruptions in motor, sensory, and autonomic functions, as well as systemic health complications (Sweis and Biller, 2017). Traumatic SCI is the second leading cause of paralysis within the United States (Armour et al., 2016), placing a lifelong burden on patients. Traumatic SCI affects over 300,000 individuals, with an incidence rate of approximately 54 cases per one million, equating to about 18,421 new cases each year (National Spinal Cord and Injury Statistical Center, 2025).

The pathophysiology of SCI consists of two distinct components: primary and secondary injuries, both of which contribute to the extent of injury severity. The primary injury is the initial mechanical damage to the cord, resulting in nerve and tissue damage at the site of injury (Ahuja et al., 2017). In contrast, the secondary injury is driven by the pathological processes that unfold following the initial insult. These include ischemia, excitotoxicity, oxidative stress, blood-spinal cord barrier breakdown and a sustained neuroinflammatory response. Together, these mechanisms amplify the extent of tissue damage, promote ongoing neurodegeneration, and shape long-term functional outcomes (Anjum et al., 2020).

Beyond these spinal processes, SCI also induces chronic systemic inflammatory consequences that contribute to long-term health complications. Persistent inflammation is a key driver of ongoing tissue pathology, immune dysfunction and neurodegeneration (Allison and Ditor, 2015; DiSabato et al., 2024; Sun et al., 2016).

While systemic inflammation is a well-known driver of SCI progression and neuroinflammation, emerging research has highlighted its impact beyond the spinal cord. The liver is a particularly important peripheral target of SCI-induced systemic inflammation. As a central hub for metabolism and immune regulation, the liver is highly susceptible to injury-induced inflammation, driving hepatic immune cell activation, cytokine production and oxidative stress (Ronca et al., 2025). Hepatic inflammation can in turn amplify systemic inflammatory responses, influencing overall recovery and contributing to multi-organ complications. Recognizing the liver as a critical target of systemic inflammation underscores the importance of identifying interventions capable of modulating these injury-induced immune responses.

While the liver represents a key site of systemic burden, the gut also plays a central role by influencing immune signaling and maintaining homeostasis through spinal cord-gut axis (Cui et al., 2023). The spinal cord-gut axis is a key contributor to systemic inflammation after SCI. The bidirectional network involves complex signaling pathways that influence inflammation, immune function and neurochemical balances (Rong et al., 2021; Strandwitz, 2018; Zhu et al., 2024). Gut microbiota and their associated metabolites are essential in modulating the spinal cord-gut axis, regulating the balance of pro- and anti-inflammatory responses (Kang et al., 2022; Kigerl et al., 2020). SCI disrupts this regulatory network, altering microbial composition and diversity (Du et al., 2021; Kong et al., 2023). These shifts are increasingly recognized as contributors to dysregulated systemic inflammation, which can affect multiple organ systems – including the liver – and influence overall SCI recovery and prognosis (Jing et al., 2023).

One pathway of particular interest that has been shown to be significantly dysregulated following SCI is the tryptophan (Trp) pathway (Du et al., 2021; Huang et al., 2023). Trp metabolism is closely linked to the gut microbiome and is crucial in numerous biological processes, including immune regulation, inflammation and neurotransmitter release (Roth et al., 2021). In addition to its systemic effects, Trp metabolism is critical for liver function. The liver is a central hub for amino acid metabolism and Trp derived metabolites can influence hepatic inflammation and fibrotic responses (Dejong et al., 2007). Dysregulation of Trp metabolism after injury may therefore contribute to hepatic dysfunction, highlighting the potential of Trp-derived metabolites to protect both central and peripheral tissues.

Microbial communities within the gastrointestinal tract convert Trp into a diverse set of bioactive metabolites, including indole and indole-3-propionic acid (IPA). These metabolites are becoming increasingly recognized for their roles in regulating systemic inflammation (Su et al., 2022; Ye et al., 2022), a crucial factor in SCI and other inflammatory diseases. IPA is widely recognized for its antioxidant and anti-inflammatory properties and consistently supports system health. In contrast, the effects of indole are more context dependent. While high concentrations or pathogen-associated sources of indole have been associated with detrimental effect, multiple studies demonstrate that host-associated, microbiota-derived indole exerts anti-inflammatory and immunomodulatory actions (Kumar et al., 2021; Ma et al., 2020). Both IPA and indole contribute to immune regulation and maintenance of gut barrier integrity, helping to reduce levels of pro-inflammatory cytokine and prevent bacterial translocation into the bloodstream (Li et al., 2021; Konopelski and Mogilnicka, 2022; Zhao et al., 2019). IPA additionally acts as a powerful antioxidant (Negatu et al., 2020) and has neuroprotective effects (Kim et al., 2023) relevant to recovery from CNS injury.

Given that SCI disrupts Trp metabolism and alters the production of key gut-derived metabolites, targeted Trp supplementation may offer a promising strategy to limit injury-induced systemic and local inflammatory pathologies. While treatments aimed at restoring balance through supplementation are not a novel approach (Che et al., 2023; Wert et al., 2020; Williams and Cao, 2024), their application in the context of central nervous system (CNS) injuries, particularly SCI, remain underexplored. This study aims to evaluate the roles of indole and IPA in modulating inflammation at both acute and chronic stages of SCI, with the goal of determining whether gut-derived metabolites can serve as therapeutic agents to mitigate injury-induced inflammatory pathologies and support recovery outcomes.

## 2 Materials and methods

### 2.1 Animal care and experimental groups

All experimental animal procedures were carried out according to the guidelines approved by the Texas A&M University Institutional Animal Use and Care Committee (IACUC, 2021-0025). 12-week-old female C57BL/6J (Jackson Laboratory, strain 000664) were randomly assigned to experimental groups and all outcome measures were assessed in a double-blinded manner. Mice were group housed up to five mice per cage in a climate-controlled facility in ventilated cages in a 12-h light/dark cycle. All mice were fed a controlled diet (Envigo, diet 8604) with ad libitum access to water.

### 2.2 Spinal cord injury

A severe contusion, compression (50kD, 2-sec dwell) injury was used in this study, similar to what described previously (Ghnenis et al., 2022), as the primary aim was to evaluate metabolic dysfunction following SCI. This injury severity reliably results in (1) locomotor deficits, (2) marked alterations in body composition, (3) hepatic dysfunction – features that parallel those observed in human SCI and (4) provides high survival. Moreover, because most human SCI cases involve mixed contusion–compression mechanisms, this model offers strong translational relevance.

Mice were assigned to either an acute (7 days post-injury, dpi) or chronic (42 dpi) cohort. For the acute, mice were randomly assigned to sham (n = 5), SCI Control (n = 6), SCI with indole treatment (SCI + Indole, n = 6) or SCI with IPA treatment (SCI + IPA, n = 6). During the 7-day period, one mouse from the SCI Control group and one mouse from the SCI + Indole group died from unknown causes, resulting in final acute cohort group sizes of n = 5 for sham, n = 5 for SCI Control, n = 5 for SCI + Indole, and n = 6 for SCI + IPA. For the chronic, mice were randomly assigned to sham (n = 7), SCI Control (n = 11), SCI + Indole (n = 11) or SCI + IPA (n = 11). Across the 42-day period, one SCI mouse per treatment group died from unknown causes, resulting in final chronic cohort sizes of n = 7 for sham and n = 10 for all SCI groups. Mice were anesthetized with 2.5 % isoflurane inhalation and a dorsal laminectomy at thoracic-8 (T8) level was done to expose the spinal cord. The Infinite-Horizon Impactor (IH-400) was used to induce a severe contusion, compression (50kD, 2-sec dwell) injury. The muscles were sutured and skin closed with Vetbond. Sham mice received laminectomy only. After surgery, all mice received daily subcutaneous injections of saline (0.9 %, MWI Veterinary Supply), buprenorphine (0.05 mg/kg, Par Pharmaceutical Chestnut Ridge, NY), and penicillin (5 mg/kg/day, Bayer Healthcare LLC, Animal Health Division Shawnee Mission, KS) for three days for hydration, pain and to prevent secondary infection, respectively. Mice were monitored daily, and their bladder expressed manually twice per day until the mice were able to urinate without assistance or until the end of the study.

### 2.3 Drug formulation and treatment

Beginning 4-h post-injury, mice in both acute and chronic cohorts received daily oral gavage drug treatments at 10 a.m. (±1 h), administered at the same time each day until the endpoint of each cohort. Indole (I3408, Sigma-Aldrich) and IPA (57400, Sigma-Aldrich) were formulated at 50 mg/kg in a 50 % DMSO solution. Sham and SCI Control groups received DMSO only as vehicle control. Total dosage volume was calculated and adjusted daily for the first 7 days and then weekly thereon based on the subject's individual body weight. Treatment effectiveness was determined through a series of behavioral and histological analyses described throughout.

### 2.4 Body composition analysis

The body weight of each mouse was taken daily for the first 7-days post injury, then weekly thereon until the end of the study. For the chronic cohort, mouse body composition (water, lean and fat weight) was scanned at baseline prior to injury and at 14-, 28-, and 42-days post-injury as determined by an EchoMRI-100 quantitative magnetic resonance whole body composition analyzer (EchoMRI-100H, Echo Medical Systems, Houston, TX) as described (Nixon et al., 2010). Briefly, mice were placed into a thin-walled plastic cylinder with a cylindrical plastic insert to limit movement. While in the tube mice were subjected to a low-intensity electromagnetic field to measure water, lean and fat weights.

### 2.5 Locomotion behavioral assessments

Locomotor recovery of mice was assessed using the Basso Mouse Scale (BMS) and rotarod tests. All behavioral tests were conducted at the same time of day and by observers blinded to experimental groups and the same observers were used throughout the duration of the study to ensure consistency. The BMS score measures hindlimb motor movement with a scoring scale of 0 (no movement) to 9 (normal locomotion). BMS subscore measures changes in stepping and coordination with a scale of 0 (no movement) to 11 (normal locomotion) (Basso et al., 2006). Mice were placed in an open field and observed for 3 min, noting features such as ankle movements, stepping pattern, coordination, paw placement, trunk stability and tail position. BMS scores were collected for both the acute and chronic cohorts on day 2 and 7 post-injury, then weekly thereon until the end of the study.

Rotarod assesses the motor coordination and balance by measuring the subject's latency to fall off the rod. Mice were placed on the rod (TSE RotaRod Advanced) rotating at increasing speed from 5 to 45 rpm in 180 s intervals. The latency to fall (seconds) was averaged between three trials per session. Mice were acclimated to the rod through four habituation sessions the week before injury, with the final baseline measure on the fifth day. Rotarod testing was conducted only for the chronic cohort on day 2 and 7 post-injury, then weekly thereon until the end of the study. Data represented as a percent of baseline latency to fall.

### 2.6 Blood plasma cytokine measurements

Blood samples were collected from the submandibular vein into a BD® Microtainer (365985, BD) using a sterile lancet. Samples were centrifuged at 14,000×*g* for 15 min at 4 °C to collect plasma. Blood was collected immediately prior to necropsy at 7 dpi (acute cohort) and 42 dpi (chronic cohort). Plasma concentrations of IFN-γ, IL-1β, IL-6, IL-13, TNF-α, MMP9, glucagon and insulin were measured using Meso Scale Discovery (MSD) U-PLEX Custom Metabolic Group 1 Assay (K152ACM-1, Meso Scale Discovery, Rockville, MD, USA) according to the manufacturer's instructions. Plasma samples were diluted 2-fold in sample Metabolic Assay Working Solution provided in the kit per manufacturer's recommendations. Data is presented as a scores plot generated from MetaboAnalyst's principal component analysis (PCA) module to assess clustering patterns across experimental groups.

### 2.7 Hepatic gene analysis

For the chronic cohort, liver tissue was collected fresh from mice immediately prior to necropsy, flash frozen in liquid nitrogen and stored at −80 °C until isolation. Cells isolated from the liver tissue were pelleted after the completion of the respective protocol followed by the extraction of RNA immediately after. Directzol RNA micro-prep columns (R2061, Zymo) were used to extract RNA from liver cells. RNA concentration was measured using the Thermo Scientific™ NanoDrop 2000. Quantabio cDNA Synthesis kit (95047, Quanta) was used to synthesize cDNA before conducting qPCR using the Quantabio PerfeCTa® SYBR® Green FastMix® (95073, Quanta) on the ViiA7 Real Time PCR system (Life Technologies). CT was calculated for each group based on the absolute CT per primer (Supplementary Table S1) subtracted by the respective CT of the negative control to reduce background noise. Each sample was analyzed in triplicate per gene of interest. Beta-Actin (ACTB) was used as the internal housekeeping gene control to normalize gene expression. Data is presented as a scores plot resulting from PCA to assess clustering patterns across experimental groups.

### 2.8 Immunostaining and histological analysis

At the endpoint of each cohort, animals were anesthetized with Sodium pentobarbital (FataPlus®) before a transcardial perfusion with 0.1M PBS-Heparin followed by 4 % paraformaldehyde (PFA). Harvested tissues were post-fixed in 4 % PFA overnight at 4 °C, then sequentially cryopreserved in 15 % and 30 % sucrose/PBS for 24 h each at 4 °C prior to embedding in Tissue-Tek® O.C.T. Compound (625501-01, Sakura Finetek). Tissue sections were stained with their respective antibodies overnight at 25 °C. All tissue sections were imaged at 20× magnification on the Olympus VS120 Slide Scanner. Quantification of all staining was done using Quantitative Pathology & Bioimage Analysis software (QuPath v0.4.2, Scotland). Three sections per animal were used for analysis.

For the chronic cohort, liver tissue was cryosectioned at 8 μm thickness cross sections, direct mount. Sections were stained with CD68 (BioRad, 1:500) and Col1α (SantaCruz Biotechnology, 1:500) to visualize macrophages and collagen fibers, respectively. For each liver section, total tissue area was calculated, and the intensity-based threshold tool was used to calculate total area of CD68^−^and Col1α-positive stain. Data is represented as fold change proportional area normalized to Sham. Statistical outlier tests identified two outliers in the CD68 data set (one SCI Control and one SCI + IPA), resulting in n = 7 sham, n = 9 SCI Control, n = 10 SCI + Indole and n = 9 SCI + IPA. Outlier test identified four outliers in the Col1α data set (one per group), thus n = 6 sham and n = 9 for SCI Control, SCI + Indole and SCI + IPA groups.

Spinal cords from both acute and chronic cohorts were cryosectioned longitudinally at 25 μm thickness. The lesion epicenter was identified, and three representative sections were collected for histological analysis: one at the epicenter, one 100 μm medial and one 100 μm lateral. Sections were stained with CD68 (BioRad, 1:500) to visualize macrophages and GFAP (DAKO, 1:500) to visualize reactive astrocytes in both cohorts. For the chronic cohort, additional markers were used: NeuN (Biotin conjugated, Sigma-Aldrich, 1:500) to visualize neurons, SOX9 (R&D Systems, 1:500) to label astrocytic nuclei and 5HT (Immunostar, 1:500) to visualize axon projections.

For each spinal cord, the lesion center was identified and sixteen annotation boxes at 200 μm thickness were drawn centered around the lesion, defining the quantification region of interest (ROI). Batching scripts were used for cell counts, intensity, and proportional area measurements. The lesion size was measured by tracing the glial scar border labeled with GFAP surrounding a DAPI + inner region, presented as um^2^. Data presented as CD68 proportional area as a percentage, relative GFAP intensity normalized to 1.6 mm rostral, relative 5HT intensity normalized to 1.6 mm rostral, SOX9 positive cells/mm^2^, and NeuN positive cells per mm^2^. 5HT lesion data represented as proportional area as a percentage within the GFAP negative space.

For spinal cord histological analysis in the chronic cohort, n = 9 for SCI Control as one animal was discarded due to poor tissue quality. Statistical outlier tests identified one outlier in the acute cohort (SCI + IPA group) and one outlier in the chronic cohort (SCI + Indole group) in the lesion size data sets. After removing these outliers, the final group sizes were n = 5 per group for the acute cohort and n = 7 sham, n = 9 SCI Control, n = 9 SCI + Indole and n = 10 SCI + IPA for the chronic cohort.

### 2.9 Statistical Analysis

Statistical analyses were performed using GraphPad Prism 9.3.1 (GraphPad Software, San Diego, CA, USA) or MetaboAnalyst 5.0. For all behavioral analyses and spinal cord immunostaining, a two-way ANOVA with or without repeated measures, respectively, followed by Tukey's multiple comparisons test was performed using GraphPad. For liver immunostaining and spinal cord lesion size a one-way ANOVA followed by Tukey's multiple comparisons test was performed using GraphPad. Differences were considered statistically significant at p ≤ 0.05. All data presented as mean ± S.E.M.

MetaboAnalyst's Statistical Analysis one factor module was used for plasma and hepatic mRNA RT-PCR analyses. Data sets were uploaded in.csv file format, followed by a data integrity check and the multivariate PCA analysis method workflow. Statistical significances of group patterns were evaluated using PERMANOVA. Distributions were computed using the Euclidean distance based on PC1 and PC2.

## 3 Results

**Fig. 1:**
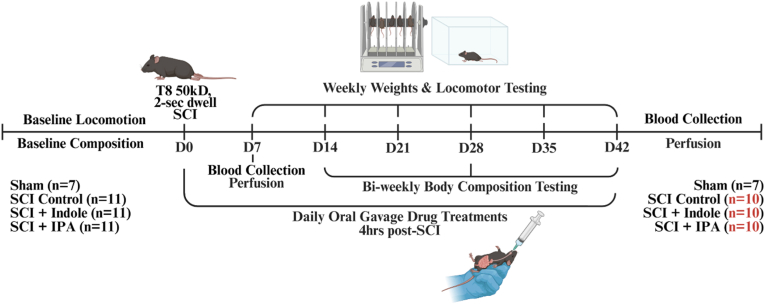
**Experimental design.** 12-week-old female C57BL/6 mice were subjected to baseline Basso Mouse Scale (BMS) and Rotarod (RR) locomotion and body composition analysis. At D0 a T8 contusion, compression (50kD, 2-sec dwell) SCI was induced. Beginning 4hrs post-injury, oral gavage drug administration began and continued daily for the duration of the study. An acute cohort of mice was perfused at 7-days. For the chromic, 42-days post-injury cohort, body weights and locomotion was assessed weekly. Body composition was assessed bi-weekly. At the end of the 42-days, blood plasma was collected followed by a transcardial perfusion and tissue harvest for histological analyses.

To assess the impact of tryptophan-derived metabolite supplementation on locomotor recovery and its efficiency in preventing systemic inflammatory pathologies after traumatic SCI, female C57BL/6J mice were subjected to a severe T8 spinal contusion-compression injury. Mice were assigned to either an acute (7 dpi) or chronic (42 dpi) cohorts, and a series of locomotor and physiological testing was conducted prior to and after injury. Beginning 4-h post-injury, respective mice were administered daily oral doses of either indole, IPA or vehicle, for the duration of their respective cohort (Fig. 1). The impacts of indole or IPA treatments on body composition, locomotion, and systemic pathologies are evaluated through a series of physiological and histological analyses, allowing assessment of both acute and longer-term treatment outcomes.

### 3.1 IPA treatment is protective against SCI-induced body composition changes

**Fig. 2:**
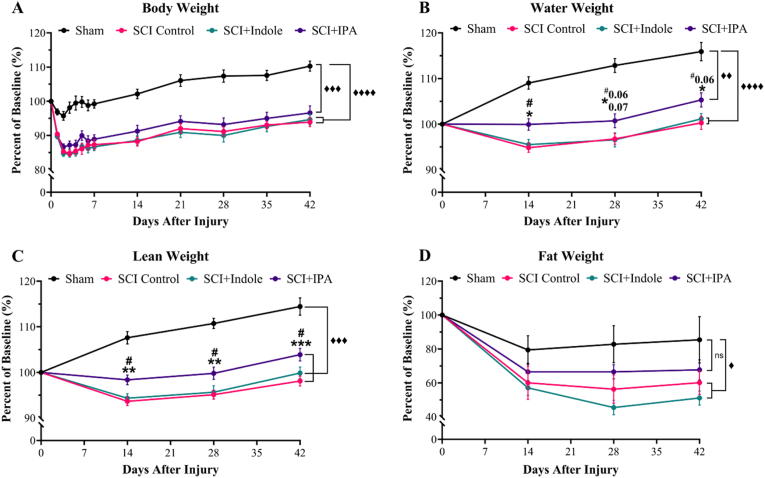
**IPA treatment prevents significant changes in body composition after spinal cord injury.** Line graphs of body weight composition represented as percent of baseline measurement. **(A)** Total body weight significantly reduced in all three SCI groups. **(B)** Water weight and **(C)** lean weight significantly reduced in SCI Control and SCI + Indole groups but remains unchanged in SCI + IPA group. SCI + IPA group significantly different than both SCI Control and SCI + Indole groups. **(D)** Fat weight significantly reduced after SCI with non-significant changes after metabolite treatments. Data presented as mean ± S.E.M.; n = 7–10. Statistical analysis performed via two-way ANOVA with repeated measures and Tukey's multiple comparisons test to determine differences between groups. ♦p < 0.05, ♦♦p < 0.01, ♦♦♦p < 0.001, ♦♦♦♦p < 0.0001 for differences between Sham and all SCI groups; ∗p < 0.05, ∗∗p < 0.01, ∗∗∗p < 0.001 for differences between SCI Control and SCI + IPA Groups; #p < 0.05 for differences between SCI + IPA and SCI + Indole groups.

Body weight measurements of each experimental subject, presented as a change from their baseline measure (Fig. 2A), show that all groups undergo a drop in body weight resulting from their respective surgical procedures. By 2 dpi, sham group stabilizes and by day 7 recovered back to their baseline levels, surpassing in weeks thereon. Conversely, all three SCI groups’ significant loss of body weight continues through day 7 (p < 0.0001). Despite a slow regain of weight throughout the duration of the 42 days, all SCI groups do not reach pre-injury baseline measures. The SCI + IPA group shows to maintain an overall higher body weight percentage than the SCI Control and SCI + Indole groups, although not statistically significant (p = 0.47 and p = 0.59, respectively). By way of an Echo-MRI, body weights are broken down into the individual components of water weight (Fig. 2B), lean weight (Fig. 2C) and fat weight (Fig. 2D). For both water and lean weight measures, the sham group increased in weight over the course of the 42 days and all three SCI groups show to be significantly less than the sham group at each measurement timepoint (p < 0.0001). However, the SCI + IPA group shows minimal deviation from its baseline measurement and is significantly different than both the SCI Control and SCI + Indole groups at each timepoint (p = 0.009 and p = 0.035, respectively). In addition, the SCI Control and SCI + Indole groups significantly dropped beginning 14 dpi with little recovery. Despite all four experimental groups showing a decrease in fat weight measurements at 14 dpi, only SCI groups show to be significantly different than their baseline measure (p = 0.002). SCI Control and SCI + Indole are significantly lower in fat weight when compared to sham (p = 0.037 and p = 0.002, respectively), while SCI + IPA shows non-significant differences from sham (p = 0.30). Overall, the lack of baseline differences within the SCI + IPA group suggests that IPA specifically has a protective effect on injury induced body composition changes.

### 3.2 IPA treatment significantly improves hindlimb locomotor recovery after injury

**Fig. 3:**
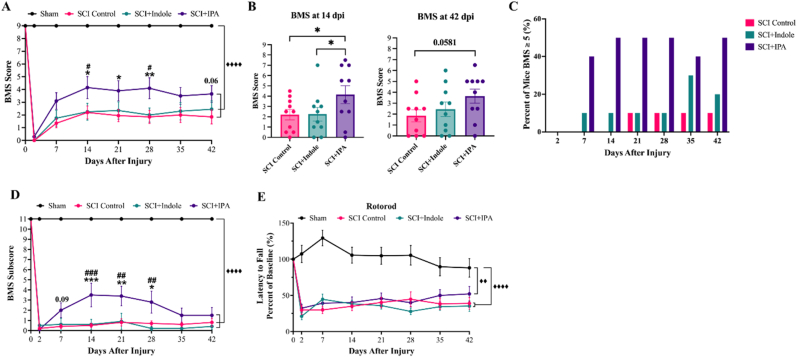
**IPA treatment significantly improves locomotor recovery after injury.** Hindlimb locomotor testing assessed using BMS and rotarod testing. **(A)** BMS scores over time; **(B)** individual BMS scores at 14 dpi and 42 dpi; **(C)** the percentage of mice achieving a BMS score ≥5; and **(D)** BMS subscores all show significant reduction in BMS scores resulting from injury, with significant improvement in SCI + IPA group beginning 14 dpi. **(E)** Rotarod latency to fall, represented as percent change from baseline shows significant decrease in total time after SCI. Data presented as mean ± S.E.M.; n = 7–10. Statistical analysis performed using one- or two-way ANOVA with repeated measures and Tukey's multiple comparisons test to determine differences between groups. ♦♦p < 0.01, ♦♦♦♦p < 0.0001 for differences between Sham and all SCI groups; ∗p < 0.05, ∗∗p < 0.01 for differences between SCI Control and SCI + IPA groups; #p < 0.05, ##p < 0.01, ###p < 0.001 for differences between SCI + IPA and SCI + Indole groups.

Locomotor function after injury was assessed at 2 dpi to confirm loss of hind limb function, then weekly thereon to monitor recovery progression through BMS scoring and Rotarod behavioral tests. BMS scores (Fig. 3A) and subscores (Fig. 3D) confirm that sham surgical procedures have no impact on hindlimb function and that all SCI surgeries inflict significant hindrance in hindlimb locomotion. At 7 dpi, all SCI groups show slight improvements in their locomotion but do remain significantly impaired when compared to sham throughout the duration of the 42 days. SCI + IPA presents non-significant differences from SCI Control and SCI + Indole groups 7 dpi (p = 0.069 and p = 0.23, respectively). Beginning 14 dpi, SCI + IPA has significantly greater BMS scores than SCI Control and SCI + Indole (p = 0.033 and p = 0.0403, respectively), which is further illustrated in the individual BMS values shown in Fig. 3B. BMS remains significantly increased at 21 and 28 dpi, loses significance at 35 dpi (p = 0.150 vs SCI Control). However, this fluctuation appears to be driven primarily by a single animal and does not represent a consistent decline at the group level. By 42 dpi (Fig. 3A and B), the group mean increases, approaching statistical significance compared with SCI Control (p = 0.0581). Interestingly, forty percent of mice treated with IPA had a BMS score greater or equal to 5 as early as 7 dpi (Fig. 3C), a score indicative of plantar stepping and occasional limb coordination, demonstrating rapid and significant functional recovery. Fifty percent of IPA treated mice reached a BMS score ≥5 by 14 dpi. Conversely, only ten percent of SCI Control mice reached a BMS score of 5 by the end of the study (42 dpi). BMS subscores show significant loss of function in all SCI groups when compared to sham (p < 0.0001), with the SCI + IPA group exhibiting significant improvements beginning 14 dpi (p = 0.0002) (Fig. 3D).

Rotarod, represented as change from baseline latency time (Fig. 3E), reveals significant decreases in all three SCI groups' latency to fall at 2 dpi when compared to sham (p < 0.0001) and is sustained throughout the duration of the 42 days. No differences are present between the three SCI groups until 35 dpi and 42 dpi, where SCI + IPA begins to show increases in latency. Taken as a whole, IPA treatments prove to have significant impact on a subject's ability to regain hindlimb locomotion, but minimal effect on coordination and balance as shown by BMS and rotarod scores, respectively.

### 3.3 The impact of metabolite treatment on systemic inflammatory markers

**Fig. 4:**
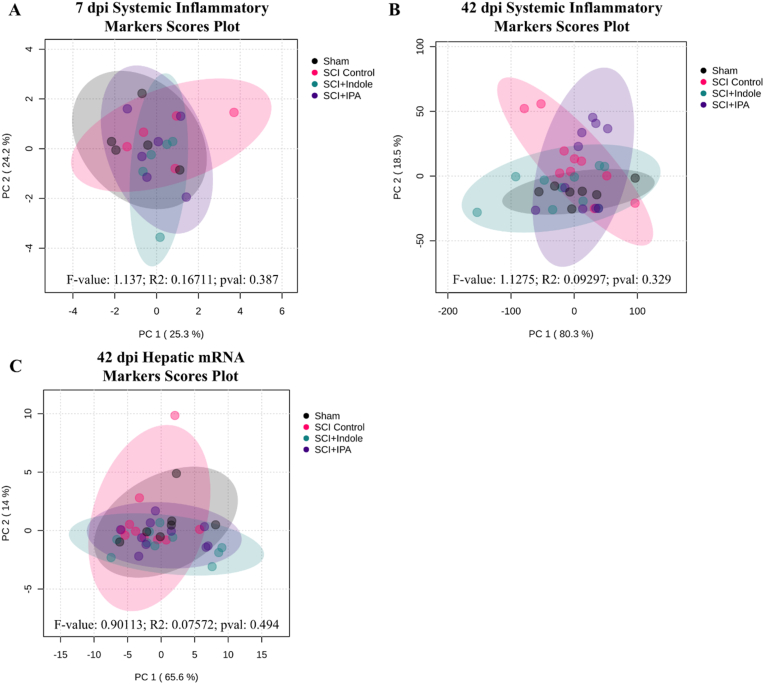
**Assessment of systemic protein and hepatic mRNA inflammatory markers at 7- and 42 days post-injury.** The principal component analysis (PCA) score scatter plot shows the distribution of circulating blood plasma protein levels at **(A)** 7- and **(B)** 42 dpi and **(C)** hepatic mRNA levels at 42 dpi for sham (black), SCI Control (pink), SCI + Indole (aqua) and SCI + IPA (purple) across the first two principal components, PC1 and PC2. The 95 % confidence interval is shown in the shaded regions for each respective group. (For interpretation of the references to colour in this figure legend, the reader is referred to the Web version of this article.)

To assess the impact metabolite treatments have on circulating inflammatory markers at 7 dpi and 42 dpi, blood plasma was collected and a series of inflammatory cytokines (IFN-γ, IL-1β, IL-6, IL-13, TNF-α, MMP9) and metabolic dysfunction markers (glucagon and insulin) were measured using a multi-plex plate-based assay. Quantifications of cytokines and metabolic markers at 7 dpi and 42 dpi showed no statistically significant group differences, indicating that systemic inflammatory profiles remained comparable across sham, SCI Control and metabolite treated groups. Individual analyte concentration plots for 7 dpi and 42 dpi plasma samples can be found in Supplementary Figs. S1 and S2, respectively. To better understand the relationship of all markers in a multidimensional manner, PCA analyses were conducted and the resulting scores plots show the distribution of circulating analyte levels at 7 dpi (Fig. 4A) and 42 dpi (Fig. 4B). Consistent with individual analyte data, PERMANOVA analyses of the PCA distributions detected no group separations, suggesting that neither injury nor metabolite treatment altered the overall composition of circulating markers. PERMANOVA p-values for all group comparisons at 7 dpi and 42 dpi are provided in Supplemental Tables S2 and S3, respectively.

### 3.4 Hepatic histological, but not transcriptional, inflammatory pathology increases after SCI and is attenuated with metabolite treatments

To determine whether metabolite supplementation following injury can mediate injury-induced liver dysfunction and inflammation, a series of hepatic inflammation (TGFβ, IL-1β, IL-6, IL-10, and TNF-α) and fibrotic markers (Col1α1, αSMA and TIMP1) were assessed at the mRNA level. No significant changes were observed in any of the experimental groups; individual analyte plots are provided in Supplementary Fig. S3. To evaluate whether subtle coordinated changes might occur among all experimental groups, PCA was conducted. Scores plot (Fig. 4C) shows substantial overlap among experimental groups and PERMANOVA analyses confirms no statistically significant multivariate differences (Supplementary Table S4). These findings indicate that by 42 dpi inflammatory and collagen related mRNA expression in the liver remain largely unchanged across groups.

**Fig. 5:**
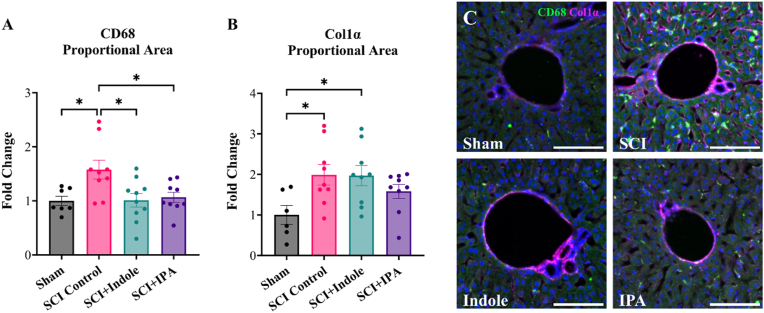
**Metabolite treatment reduces injury-induced liver inflammation and fibrosis. (A)** Quantification of CD68 proportional area, represented as fold change, shows significant increase in SCI Control group and a significant reduction in both SCI + Indole and SCI + IPA groups. **(B)** Quantification of Col1α proportional area, represented as fold change, shows a significant increase in SCI Control and SCI + Indole groups. **(C)** Representative images of liver sections immunolabeled for CD68 (green), Col1a (magenta) and DAPI (blue). Images shown at 20× magnification. Scale bar = 100 μm. Data presented as mean ± S.E.M.; n = 7–10. Statistical analysis performed using one-way ANOVA with Tukey's multiple comparisons test to determine differences between groups. ∗p < 0.05. (For interpretation of the references to colour in this figure legend, the reader is referred to the Web version of this article.)

Immunostaining was used to examine the expression and distribution of CD68 (Fig. 5A) and Col1α (Fig. 5B) within the liver at 42 dpi. Following SCI, CD68 proportional area is significantly increased in comparison to the sham group (p = 0.026). Both treatments of indole and IPA significantly decrease the expression of CD68 in comparison to the SCI Control (p = 0.015 and p = 0.037, respectively), restoring expression back to sham levels. Similarly, Col1α is shown to be significantly increased in SCI Control group (p = 0.041). Indole treatments reveal to have no effect on Col1α expression, remaining significantly increased in comparison to sham (p = 0.0467). IPA treated mice display Col1α levels that lie between those of sham and SCI Control groups, with no statistically significant differences from either group. CD68^+^ macrophages were present and distributed evenly throughout the liver sections, whereas Col1α deposits were concentrated at and around the central hepatic triad (Fig. 5C).

### 3.5 Metabolite treatment reduces local inflammation, but has no significant impact on astrocyte, neuron and axon populations within the spinal cord after injury

**Fig. 6:**
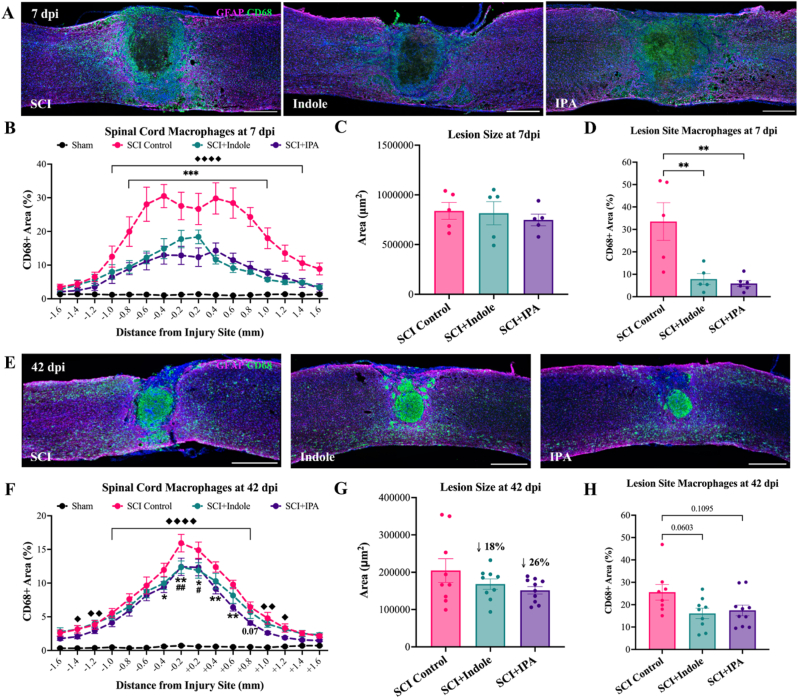
**Metabolite treatment significantly reduces injury-induced inflammation within the spinal cord.** Acute cohort, 7 dpi: **(A)** Representative longitudinal sections of the spinal cord, **(B)** quantification of spinal cord CD68 proportional area (%), **(C)** lesion size (μm^2^), **(D)** CD68 proportional area (%) within the lesion. Chronic cohort, 42 dpi: **(E)** representative longitudinal sections of the spinal cord, **(F)** quantification of spinal cord CD68 proportional area (%), **(G)** lesion size (μm^2^), and **(H)** CD68 proportional area (%) within the lesion. Spinal cords immunolabeled with CD68 (green), GFAP (magenta) and DAPI (blue). Images shown at 20× magnification. Scale bar = 500 μm. Data presented as mean ± S.E.M.; n = 7–10. Statistical analysis performed using one- or two-way ANOVA with Tukey's multiple comparisons test to determine differences between groups. ♦p < 0.05, ♦♦p < 0.01, ♦♦♦♦p < 0.0001 for differences between Sham and all SCI groups. ∗p < 0.05, ∗∗p < 0.01, ∗∗∗p < 0.001 for differences between SCI Control and SCI + IPA. #p < 0.05, ##p < 0.01 for differences between SCI Control and SCI + Indole. (For interpretation of the references to colour in this figure legend, the reader is referred to the Web version of this article.)

To evaluate the effect of metabolite treatment on inflammation within the spinal cord, tissue sections were stained with CD68 at 7 dpi (Fig. 6A) and 42 dpi (Fig. 6E). At 7 dpi, quantification of CD68 proportional area shows significant increases in the SCI Control group compared to sham (p < 0.0001), while treatment with indole or IPA significantly reduces CD68 positive area from 0.8 mm rostral of the injury site and continuing through 1.0 mm caudal of injury site (Fig. 6B). Detailed p-values for each group comparison at each distance along the spinal cord are provided in Supplementary Table S5. Lesion size at 7 dpi does not differ between control and treatment groups (Fig. 6C). However, within the lesion site, both indole and IPA treatments significantly decrease macrophage accumulation compared to the SCI Controls (p = 0.0073 and p = 0.0031, respectively) (Fig. 6D). At 42 dpi, CD68 immunoreactivity remains elevated in SCI Controls, indicating sustained inflammation. Quantifications confirm significant increases in CD68 proportional area in all three SCI groups relative to sham (p < 0.0001, Fig. 6F). A significant reduction of CD68 within the SCI + Indole group is seen ±0.2 mm from the injury site. SCI + IPA shows a significant reduction in CD68 area at 0.4 mm rostral of the injury site and continuing through 0.6 mm caudal of injury site. Detailed p-values for each group comparison at each distance along the spinal cord are provided in Supplementary Table S6. Lesion size was modestly reduced at 42 dpi in the indole and IPA treated groups by 18 and 26 percent, respectively, though these differences were not statistically significant (Fig. 6G). Assessment of CD68 macrophage area specifically within the lesion (Fig. 6H) shows trends toward decreased accumulation in indole and IPA treated groups (p = 0.0603 and p = 0.1095, respectively). In summary, metabolite treatment attenuates early and persistent inflammation in the injured spinal cord, reflected by reduced macrophage accumulations both within and along the surrounding tissue.

**Fig. 7:**
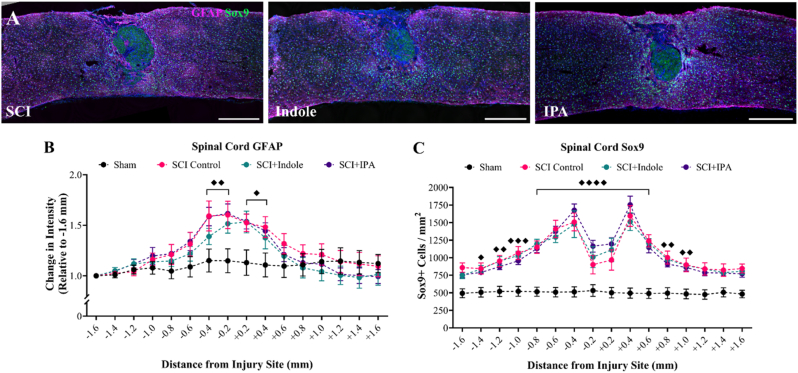
**Effect of metabolite treatment on astrogliosis within the spinal cord. (A)** Representative longitudinal sections of the spinal cord immunolabeled with GFAP (magenta), Sox9 (green) and DAPI (blue). Images shown at 20× magnification. Scale bar = 500 μm. **(B)** Quantification of spinal cord GFAP shows significant increases in all SCI groups. **(C)** Quantification of spinal cord Sox9 shows significant increases in all SCI groups. Data presented as mean ± S.E.M.; n = 7–10. Statistical analysis performed using two-way ANOVA with Tukey's multiple comparisons test to determine differences between groups. ♦p < 0.05, ♦♦p < 0.01, ♦♦♦p < 0.001, ♦♦♦♦p < 0.0001 for differences between Sham and all SCI groups. (For interpretation of the references to colour in this figure legend, the reader is referred to the Web version of this article.)

Assessing changes in astrogliosis after injury and treatment is evaluated through GFAP and Sox9 immunostaining (Fig. 7A). A 1.5-fold increase in GFAP intensity ±0.4 mm from the injury site is seen resulting from SCI (Fig. 7B). SCI + Indole and SCI + IPA groups show no significant changes compared to SCI Control group in intensity. Detailed p-values for each group comparison at each distance along the spinal cord are provided in Supplementary Table S7. Furthermore, the total number of Sox9+ cells within the cord were analyzed (Fig. 7C). Both SCI + Indole and SCI + IPA groups exhibited elevated counts surrounding the injury site relative to SCI Control group, although these increases did not statistically differ from SCI Control (at −0.2 mm p = 0.11, +0.2 mm p = 0.21). Detailed p-values for each group comparison at each distance along the spinal cord are provided in Supplementary Table S8. Moreover, these results infer that despite minimal changes in overall intensity of astrocytes, there is an increase in the overall number surrounding the injury site as determined by the astrocytic-nuclear marker Sox9.

**Fig. 8:**
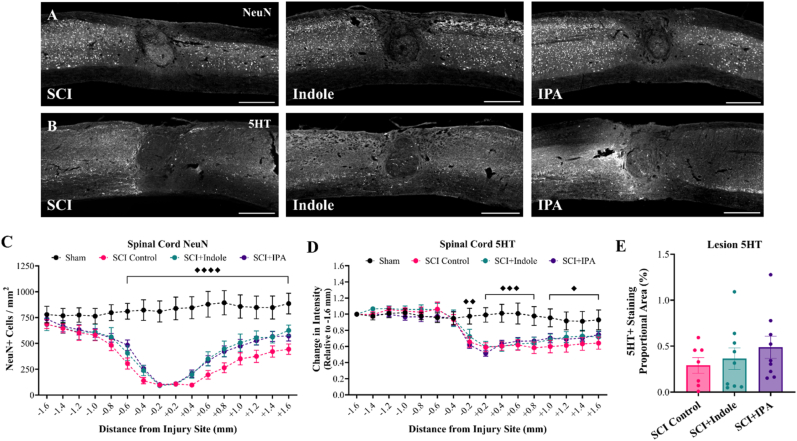
**Effect of metabolite treatment on neuronal and axonal populations within the spinal cord. (A, B)** Representative longitudinal sections of the spinal cord immunolabeled with **(A)** NeuN and **(B)** 5HT. Images shown at 20× magnification. Scale bar = 500 μm. **(C)** Quantification of spinal cord NeuN shows significant decreases in SCI Control group, with non-significant changes in SCI + Indole and SCI + IPA groups. **(D)** Quantification of spinal cord 5HT shows significant decreases in SCI Control group. **(E)** Quantification of 5HT proportional area (%) within the lesion site shows minimal increases in SCI + Indole and SCI + IPA groups. Data presented as mean ± S.E.M.; n = 7–10. Statistical analysis performed using two-way ANOVA with Tukey's multiple comparisons test to determine differences between groups. ♦p < 0.05, ♦♦p < 0.01, ♦♦♦p < 0.001, ♦♦♦♦p < 0.0001 for differences between Sham and all SCI groups.

Neuron and axon populations in the cord were assessed through NeuN (Fig. 8A) and 5HT (Fig. 8B) immunostaining as measured by total number of NeuN + cells (Fig. 8C) and intensity (Fig. 8D), respectively. Quantifications show a significant reduction in the number of NeuN + cells following SCI (p < 0.0001), with elevated cell counts in both SCI + Indole (at +0.6 mm p = 0.24, +0.8 mm p = 0.12, +1.0 mm p = 0.24, +1.2 mm p = 0.13, +1.6 mm p = 0.12) and SCI + IPA (at +0.6 mm p = 0.33, +0.8 mm p = 0.20, +1.2 mm p = 0.23, +1.4 mm p = 0.27) groups beginning 0.6 mm caudal of the injury site. Detailed p-values for each group comparison at each distance along the spinal cord are provided in Supplementary Table S9. 5HT staining revealed significant reductions in staining intensity in all three SCI groups beginning 0.2 mm caudal of injury site and continuing throughout the ROI, with no differences in either SCI + Indole or SCI + IPA groups. Detailed p-values for each group comparison at each distance along the spinal cord are provided in Supplementary Table S10. Additionally, both treatments exhibited no effect on the proportional area of 5HT staining within the lesion core (Fig. 8E).

## 4 Discussion

The present study aimed to evaluate the effects on indole and IPA on the recovery and pathophysiological changes following SCI. The key findings indicate several significant outcomes regarding body composition, locomotor function, hepatic and local inflammation. First, IPA demonstrates to be protective against injury-induced body composition changes, suggesting its role in preserving metabolic balance after SCI. Second, IPA treatment leads to notable improvements in hindlimb locomotor recovery, highlighting its potential as a therapy for enhancing recovery post-injury. Additionally, both indole and IPA treatments significantly reduce liver inflammation. Within the spinal cord, both treatments reduce injury-induced inflammation at both the acute and chronic stages, but demonstrate no significant effects on astrocytic, neuronal, and axonal populations following injury.

### 4.1 Systemic effects of SCI on body composition and liver function

Significant changes in body composition following SCI are observed in both human and pre-clinical rodent models. In humans, changes are primarily characterized by decreases in lean muscle mass and increases in fat accumulation (Bauman and Spungen, 2000), likely resulting from the loss of motor control and reduction of physical activity. Our results demonstrate significant reductions in all composition measures following injury, consistent with other studies of varying injury models (Ghnenis et al., 2022; Harris et al., 2019; Primeaux et al., 2007). Independent of the species-specific responses, the trauma induced composition changes are likely a result of impaired metabolic function (Gorgey et al., 2014). Our study demonstrates that IPA plays a significant role in preventing injury-induced changes, highlighting its potential in maintaining metabolic homeostasis. Notably, these protective effects were observed in the absence of significant alterations in circulating inflammatory cytokines, suggesting the systemic inflammation alone is unlikely to fully account for IPA's effects.

IPA may exert its protective effects on body composition through several alternate mechanisms, including modulation of gut microbiota composition (Zhao et al., 2019), enhancement of intestinal integrity or local tissue-specific actions in muscles and adipose tissue which can aid in restoring proper metabolic balance (Li et al., 2024). Additionally, IPA's antioxidant properties (Negatu et al., 2020) and potential to enhance glucose metabolism and insulin sensitivity (Zhang et al., 2022) could contribute to maintaining lean mass and preventing fat accumulation after SCI. These findings indicate that IPA may protect body composition through mechanisms that are independent of systemic cytokine changes, warranting further investigation into local and central metabolic mechanisms.

SCI triggers systemic inflammatory responses, leading to elevated levels of pro-inflammatory cytokines in the bloodstream (Hellenbrand et al., 2021; Jaffer et al., 2010). While the primary injury occurs in the spinal cord, the systemic consequences of SCI significantly impact the peripheral organs, particularly the liver, which is highly sensitive to inflammatory stress. Research has shown that chronic systemic inflammation significantly affects liver health, serving as a key driver in the progression of liver diseases, including liver fibrosis and damage (Simbrunner et al., 2022; Tanwar et al., 2020), due to the activation of inflammatory pathways within the liver tissue. Together, SCI and these inflammatory processes create a vicious cycle, where liver dysfunction not only exacerbates SCI-related pathologies, but may also influence recovery and secondary complications (Goodus et al., 2021).

Research indicates that indole and IPA have protective effects on liver function in SCI-independent models (Hendrikx and Schnabl, 2019; Ma et al., 2020; Zhao et al., 2019). In the context of SCI, these effects may be particularly relevant, as preserving liver function could mitigate inflammation and improve overall outcomes. Indole mitigates inflammation and alleviates the progression of liver diseases through its anti-inflammatory properties and interaction with the aryl hydrocarbon receptor (AhR) pathway (Wang et al., 2022; Xu et al., 2021). In addition to binding to AhR, IPA interacts with the pregnane X receptor (PXR), a key regulator of liver metabolism and detoxification (Sehgal et al., 2022; Zhao et al., 2024). During liver inflammation, AhR and PXR are dysregulated, leading to impaired detoxification and increased liver damage (Carambia and Schuran, 2021; Sayaf et al., 2021). However, the addition of tryptophan-derived metabolites has been shown to induce activation of these receptors (Wang et al., 2022, 2023; Wrzosek et al., 2021), thereby promoting liver protection and reducing hepatic insult by restoring normal metabolic and detoxification processes. This activation of AhR and PXR by IPA and related metabolites may help reestablish liver homeostasis and indirectly influence body composition and systemic metabolic balance.

### 4.2 CNS effects: inflammation, neuronal preservation and motor recovery

Loss of motor function varies depending on injury severity and location. Behavioral assessments, such as BMS and rotarod, allow for the quantitative observation of deficits throughout injury progression. Our previous work shows that moderate to severe thoracic injury causes significant hindlimb locomotion deficits (Ghnenis et al., 2022; Hook et al., 2022) and is further confirmed in the present study. SCI Control mice show minimal recovery over the course of 42 days in both BMS and rotarod behavioral assessments. Mice in the SCI + Indole group demonstrated no improvement in either locomotion test. In contrast, subjects in the SCI + IPA group exhibited significant improvement in BMS scores beginning 14 dpi. IPA's neuroprotective and anti-inflammatory properties may help mitigate the initial damage caused by SCI, creating an environment more conducive to repair and functional recovery.

Our analysis at 7 dpi revealed that both indole and IPA treatments significantly reduced CD68^+^ macrophage infiltration into the spinal cord. This early anti-inflammatory effect suggests that both compounds can attenuate the initial innate immune response following SCI, which is critical for limiting secondary injury mechanisms. While indole's suppression of early inflammation did not translate into long-term locomotor function, IPA treatment resulted in both early inflammation reduction and sustained functional recovery, indicating additional neuroprotective mechanisms at later stages of injury. Indole and IPA have been shown to protect the blood-brain barrier (BBB) (Rosell-Cardona et al., 2025; Zhao et al., 2022), and it may similarly reduce blood-spinal cord barrier permeability after SCI. By limiting barrier leakage, IPA could decrease infiltration of peripheral immune cells into the spinal cord, thereby dampening local inflammation and enhancing neuron survival.

The antioxidant effects of indole and its derivatives (Ji et al., 2020; Negatu et al., 2020), as previously discussed, are especially relevant within the spinal cord, where oxidative stress contributes significantly to secondary injury mechanisms (Yin et al., 2024). By scavenging reactive oxygen species, IPA may help preserve neuronal integrity following SCI (Feng et al., 2023). Our analysis at 42 dpi shows a higher number of neurons in the spinal cords of IPA treated animals compared to SCI Control, although not statistically significant, suggesting that IPA may contribute to neuronal preservation. Chronic inflammation resulting from SCI exacerbates neuronal damage (Jin et al., 2023). Indole and IPA exhibit local anti-inflammatory effects, as demonstrated by the reduction of CD68^+^ macrophages in the spinal cord. CD68 is a marker of activated macrophages involved in pro-inflammatory responses, often associated with NF-kB pathway activation (Liang et al., 2022). Various indole derivates have been shown to inhibit NF-kB signaling (Ji et al., 2020; Takada et al., 2025), attenuating inflammatory cascades. In the context of SCI, early inhibition of NF-kB signaling likely contributed to the reduction of CD68^+^ macrophage infiltration at 7 dpi, mitigating inflammation and potentially protecting against further neuronal damage. Collectively, these finding suggest a temporal distinction in the effects of indole and IPA – both compounds reduce early macrophage-mediated inflammation, but only IPA induces functional locomotor functions, highlighting its therapeutic potential following SCI.

### 4.3 Axonal regeneration and astrocyte modulation

In addition to its protective effects, IPA may promote recovery by fostering axonal growth. Recent studies have demonstrated that IPA enhances axonal regeneration in the peripheral nervous system (PNS), accelerating the recovery of sensory function (Serger et al., 2022; Zhang et al., 2025). However, when assessing axon populations in the spinal cord, we observed no significant differences between the SCI Control and treated groups. This discrepancy may stem from the differing regenerative responses of the CNS and PNS. In the PNS, Schwann cells rapidly de-differentiate, proliferate and release neurotrophic factors to guide axon growth (Jessen and Mirsky, 2019). In contrast, the CNS presents both intrinsic and extrinsic barriers to regeneration, including glial scar formation (Okada et al., 2018) and myelin-associated inhibitors (Geoffroy and Zheng, 2014), which physically and chemically impede neurite outgrowth, respectively. This may suggest that IPA's role in regeneration may be system specific and its contribution to locomotor recovery might not be directly linked to axon regeneration in the spinal cord. Instead, IPA's well documented anti-inflammatory and antioxidant properties, as previously discussed, may provide indirect support for axonal regeneration by stabilizing mitochondrial function and preserving circuit-level activity (Singh et al., 2024). Nonetheless, further research is necessary to elucidate the specific effect of IPA on CNS regeneration.

IPA's ability to modulate the spinal cord environment is likely another contributing factor to promoting recovery. Following injury, astrocytes undergo an acute response, becoming reactive and forming a glial border around the lesion, which serves as a physical barrier to prevent further damage (Faulkner et al., 2004; Okada et al., 2018). Tryptophan metabolites have been shown to modulate astrocyte activity within the CNS (Rothhammer et al., 2016; Sun et al., 2022). While our data shows no effect of IPA on astrocytic expression intensity, we observed elevated counts in the astrocytic transcription marker Sox9 in regions surrounding the lesion center. Sox9 is a transcription factor critical for astrocytic development and has been shown to contribute to astrogliosis and glial scar formation (Clifford et al., 2023), helping to contain inflammation and limit the spread of secondary damage. Studies investigating the role of astrocytes in lesion development suggest that proper regulation promotes lesion containment and tissue protection (Bradbury and Burnside, 2019; Chen et al., 2025). IPA treatment reduced lesion size by 26 percent compared to SCI Control at 42dpi. While no direct mechanistic link between IPA and Sox9 has been established, IPA's immunomodulatory properties may indirectly promote Sox9 expression by influencing STAT3 signaling, a known upstream regulator of Sox9 during astrocyte activation. Previous studies have shown that IPA exerts anti-inflammatory effects on primary human astrocytes in the presence of chronic low-grade inflammation (Garcez et al., 2020). Additionally, IPA has been shown to be involved in STAT3 signaling (Hao et al., 2025; Wang et al., 2024), further supporting its potential role in modulating astrocytic activity. By reducing pro-inflammatory signals, IPA may enhance STAT3-driven transcriptional program, promoting a more protective astrocyte phenotype. In line with this, IPA has shown to influence astrocyte phenotypes, promoting a shift toward neuroprotective over neurotoxic states (Xie et al., 2022). Taken together, these findings suggest that IPA may indirectly influence Sox9-mediated responses by reducing inflammation and contributing to a more controlled glial reaction, which may help limit lesion size and support functional recovery.

## 5 Limitations

While this study demonstrates that microbiota-derived metabolites can influence recovery following SCI, there are a few limitations that must be acknowledged. Although we observed associations between metabolite treatment and improved locomotor function, systemic and organ-specific mechanistic links remain unclear. The PCA of plasma and hepatic mRNA expression did not yield statistically significant group differences, limiting our ability to definitively attribute functional improvements to systemic inflammatory modulation. Additionally, while IPA treatment consistently outperformed indole in functional and histological outcomes, neither metabolite affected inflammatory profiles. This dissociation suggests that systemic inflammation may not be the sole driver of recovery and highlights the complexity of linking molecular changes to functional outcomes. Future studies incorporating broader molecular profiling and cell-type specific analyses will be important to capture the complexity of tissue responses and to further elucidate the distinct roles of these metabolites across the spinal cord-gut-liver axis.

Female mice were used exclusively in this study to reduce biological variability and avoid potential confounding effects of sex as a biological variable. Prior studies have demonstrated sex-specific differences in injury response, immune activation, and locomotor recovery that can obscure treatment effects when both sexes are combined. Focusing on a single sex allowed for greater consistency in outcomes and improved statistical power to detect treatment-related changes. Female mice also offer practical advantages, including easier handling, fewer post-injury urinary complications, and lower mortality. Future studies will include male mice to evaluate potential sex-specific responses to treatment.

The use of daily oral gavage can introduce variability due to minor inconsistencies in technique (e.g., depth, speed, reflux, or minor esophageal irritation) (Taylor et al., 2025). Handling and recent gavage may transiently influence locomotor performance, making it challenging to fully separate drug effects from procedural effects. Stress associated with repeated gavage can also modulate cytokine production and gut–immune signaling, meaning that fluctuations in inflammation profiled could reflect interactions between drug and stress rather than drug action alone. Alternative delivery methods were considered: intraperitoneal injections may be equally stressful and potentially less effective, while drug administration via food or water would minimize stress but make it difficult to ensure uniform dosing and would result in continuous rather than once-daily exposure. To mitigate potential confounds, all gavage procedures were performed by the same individual and at the same time of day to reduce variability, dosing was conducted after locomotor and behavioral testing to minimize acute performance effects, and both uninjured and injured control groups received daily gavage to control for procedural influences. These steps help ensure that the observed outcomes largely reflect the effects of the drug while acknowledging the inherent limitations of the administration method.

## 6 Conclusion

In summary, this research represents an innovative effort to explore metabolite supplementation as a treatment for inflammatory pathologies following SCI. Our findings indicate that tryptophan-derived metabolite supplementation enhances functional recovery following SCI and attenuates local inflammatory pathologies in the liver and spinal cord, likely by modulation of metabolic and pro-inflammatory responses, even though systemic inflammatory profiles remain largely unchanged. Future studies on investigating the cellular and molecular mechanisms by which indole and IPA influence liver function will be important to clarify their contribution to hepatic inflammation and metabolic regulation. Additionally, assessment of metabolite supplementation in SCI-induced gut dysfunction will be essential to fully understand their therapeutic potential, particularly in modulating colon function, inflammation, and the composition of microbial and metabolite populations. Direct assessment of intestinal barrier integrity - such as gut permeability assays and analysis of tight junction protein expression - will be important to determine whether metabolite treatments exert protective effects through the restoration of intestinal barrier function. Advancing our knowledge of tryptophan metabolites’ therapeutic potential could pave the way for natural-based therapeutic strategies – not only for individuals with SCI, but for other CNS diseases characterized by similar inflammatory processes.

## CRediT authorship contribution statement

**Ashley J. Douthitt:** Conceptualization, Data curation, Formal analysis, Methodology, Project administration, Visualization, Writing – original draft, Writing – review & editing. **Aaron Bennett:** Formal analysis, Investigation, Visualization, Writing – original draft. **Alexandra Koustova:** Formal analysis, Investigation, Visualization, Writing – original draft. **Asim Abdelfattah:** Investigation. **Darijana Horvat:** Investigation. **Cédric G. Geoffroy:** Conceptualization, Formal analysis, Funding acquisition, Investigation, Methodology, Project administration, Resources, Supervision, Validation, Visualization, Writing – original draft, Writing – review & editing.

## Funding

This work was generously supported by the 10.13039/100015610Windermere Foundation.

## Declaration of competing interest

The authors declare that they have no known competing financial interests or personal relationships that could have appeared to influence the work reported in this paper.

## Data Availability

Data will be made available on request.
