# Supplementary material for: Leveraging microbiota-metabolites to reduce inflammation and promote functional recovery following spinal cord injury in female mice
Source: Brain Behav Immun Health. 2025 Dec 2;50:101157. doi: 10.1016/j.bbih.2025.101157 (PMC12719675; doi:10.1016/j.bbih.2025.101157)
Supplement: Multimedia component 1 [file mmc1.docx]

**Supplementary Data**

**Supplementary Table S1. Primers for Real-Time Polymerase Chain Reaction based quantification of hepatic gene expression in mice.**

| Name | Forward Sequence | Reverse Sequence |
| --- | --- | --- |
| Col1α1 | GCTCCTCTTAGGGGCCACT | CCACGTCTCACCATTGGGG |
| αSMA | GTTCAGTGGTGCCTCTGTCA | ACTGGGACGACATGGAAAAG |
| TIMP1 | AGGTGGTCTCGTTGATTTCT | GTAAGGCCTGTAGCTGTGCC |
| TGFβ | GTGGAAATCAACGGGATCAG | ACTTCCAACCCAGGTCCTTC |
| IL-1β | GCAACTGTTCCTGAACTCAACT | ATCTTTTGGGGTCCGTCAACT |
| IL-6 | TAGTCCTTCCTACCCCAATTTCC | TTGGTCCTTAGCCACTCCTTC |
| IL-10 | GCTCTTACTGACTGGCATGAG | CGCAGCTCTAGGAGCATGTG |
| TNFα | CCCTCACACTCAGATCATCTTCT | GCTACGACGTGGGCTACAG |
| ACTB | CTCTGGCTCCTAGCACCATGAAGA | GTAAAACGCAGCTCAGTAACAGTCCG |

Col, collagen; SMA, smooth muscle actin; TIMP, tissue inhibitor of metalloproteinases; TGF, transforming growth factor; IL, interleukin; TNF, tumor necrosis factor; ACTB, beta Actin.

**
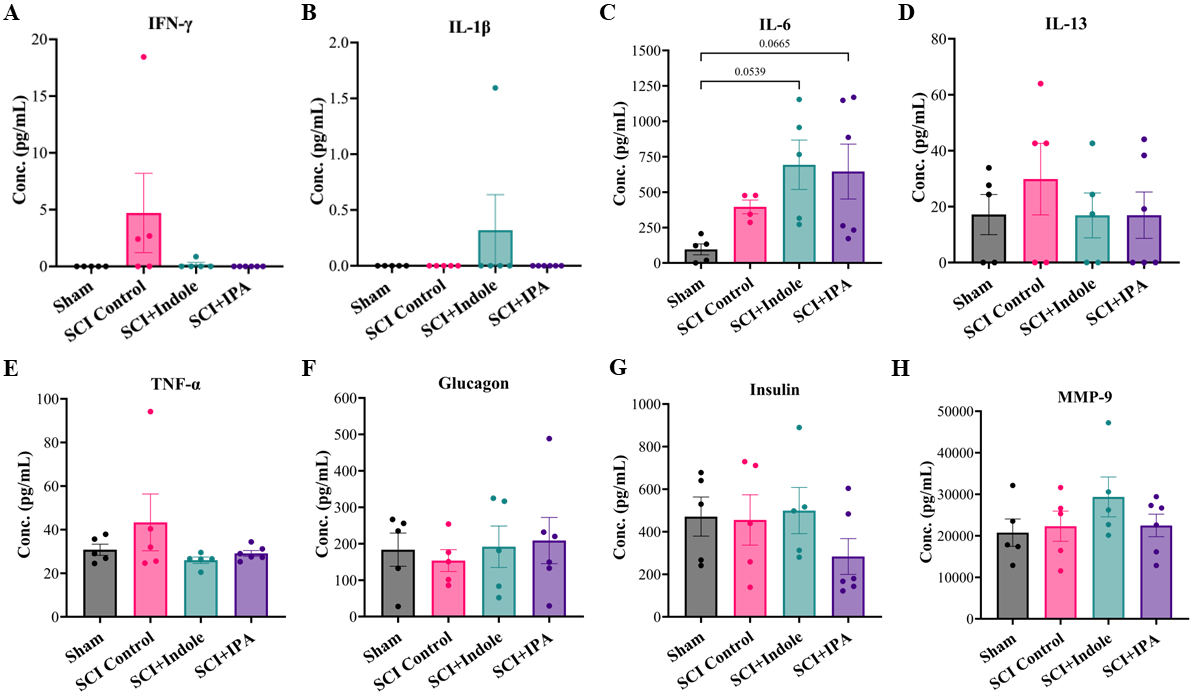
**

**Supplementary Figure S1. Circulating blood plasma protein concentration (pg/mL) levels at 7 dpi measured via MSD Custom Biomarker U-PLEX Assay platform**. (A) IFN-γ, (B) IL-1β, (C) IL-6, (D) IL-13, (E) TNF-α, (F) Glucagon, and (G) Insulin, (H) MMP9. No statistical differences in circulating levels in SCI or treatment groups. Data presented as mean ± S.E.M.; n=4-6. Data analyzed by one-way ANOVA with Tukey’s multiple comparisons test.

**
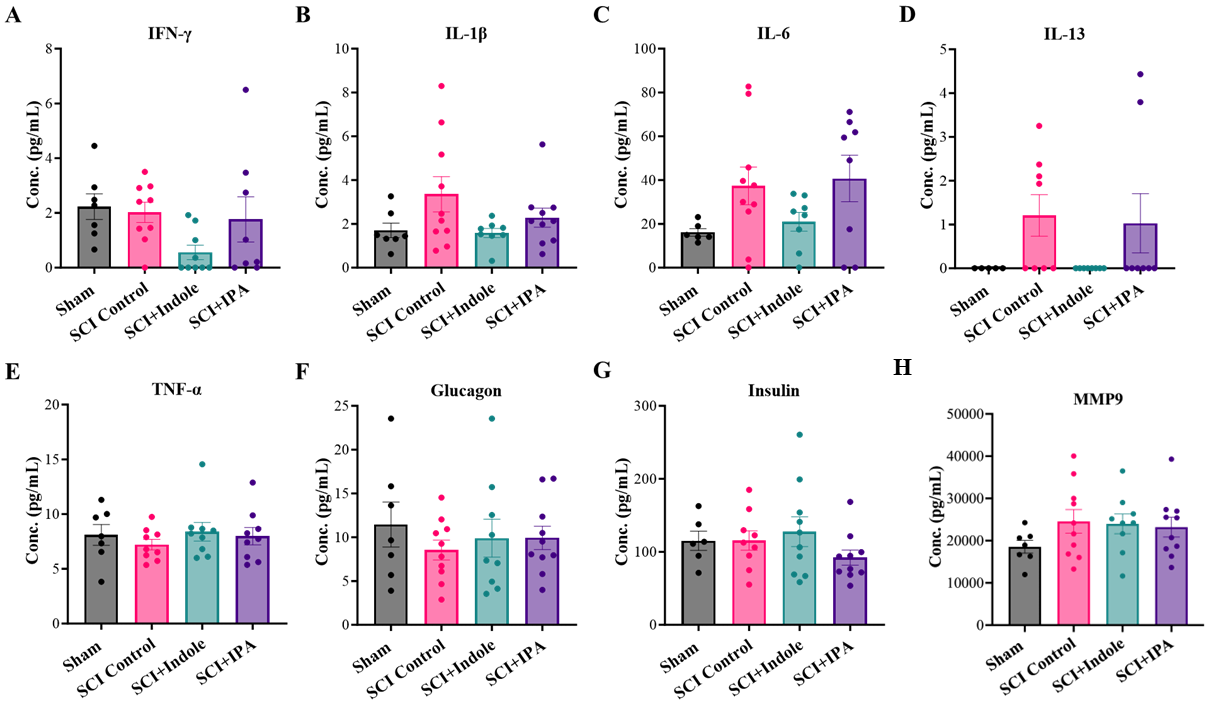
**

**Supplementary Figure S2. Circulating blood plasma protein concentration (pg/mL) levels at 42 dpi measured via MSD Custom Biomarker U-PLEX Assay platform**. (A) IFN-γ, (B) IL-1β, (C) IL-6, (D) IL-13, (E) TNF-α, (F) Glucagon, and (G) Insulin, (H) MMP9. No statistical differences in circulating levels in SCI or treatment groups. Data presented as mean ± S.E.M.; n=6-10. Data analyzed by one-way ANOVA with Tukey’s multiple comparisons test.

**Supplementary Table S2. Pairwise PERMANOVA results of 7 dpi plasma inflammatory markers.**

|  | **F.Model** | **R2** | **pval** | **p.adj** |
| --- | --- | --- | --- | --- |
| SCI Control vs SCI+Indole | 1.52560 | 0.16016 | 0.26900 | 0.53800 |
| SCI Control vs SCI+IPA | 0.57648 | 0.06020 | 0.61800 | 0.61800 |
| SCI Control vs Sham | 1.52450 | 0.16006 | 0.25700 | 0.53800 |
| SCI+Indole vs SCI+IPA | 0.61459 | 0.06392 | 0.51700 | 0.61800 |
| SCI+Indole vs Sham | 1.95740 | 0.19658 | 0.16700 | 0.53800 |
| SCI+IPA vs Sham | 0.75047 | 0.07697 | 0.47700 | 0.61800 |

**Supplementary Table S3. Pairwise PERMANOVA results of 42 dpi plasma inflammatory markers.**

|  | **F.Model** | **R2** | **pval** | **p.adj** |
| --- | --- | --- | --- | --- |
| SCI Control vs SCI+Indole | 1.22460 | 0.06370 | 0.29100 | 0.53640 |
| SCI Control vs SCI+IPA | 0.35361 | 0.01927 | 0.67400 | 0.67400 |
| SCI Control vs Sham | 0.80467 | 0.05091 | 0.38900 | 0.53640 |
| SCI+Indole vs SCI+IPA | 2.36160 | 0.11598 | 0.12100 | 0.53640 |
| SCI+Indole vs Sham | 0.87328 | 0.05502 | 0.39100 | 0.53640 |
| SCI+IPA vs Sham | 0.77682 | 0.04924 | 0.44700 | 0.53640 |

**
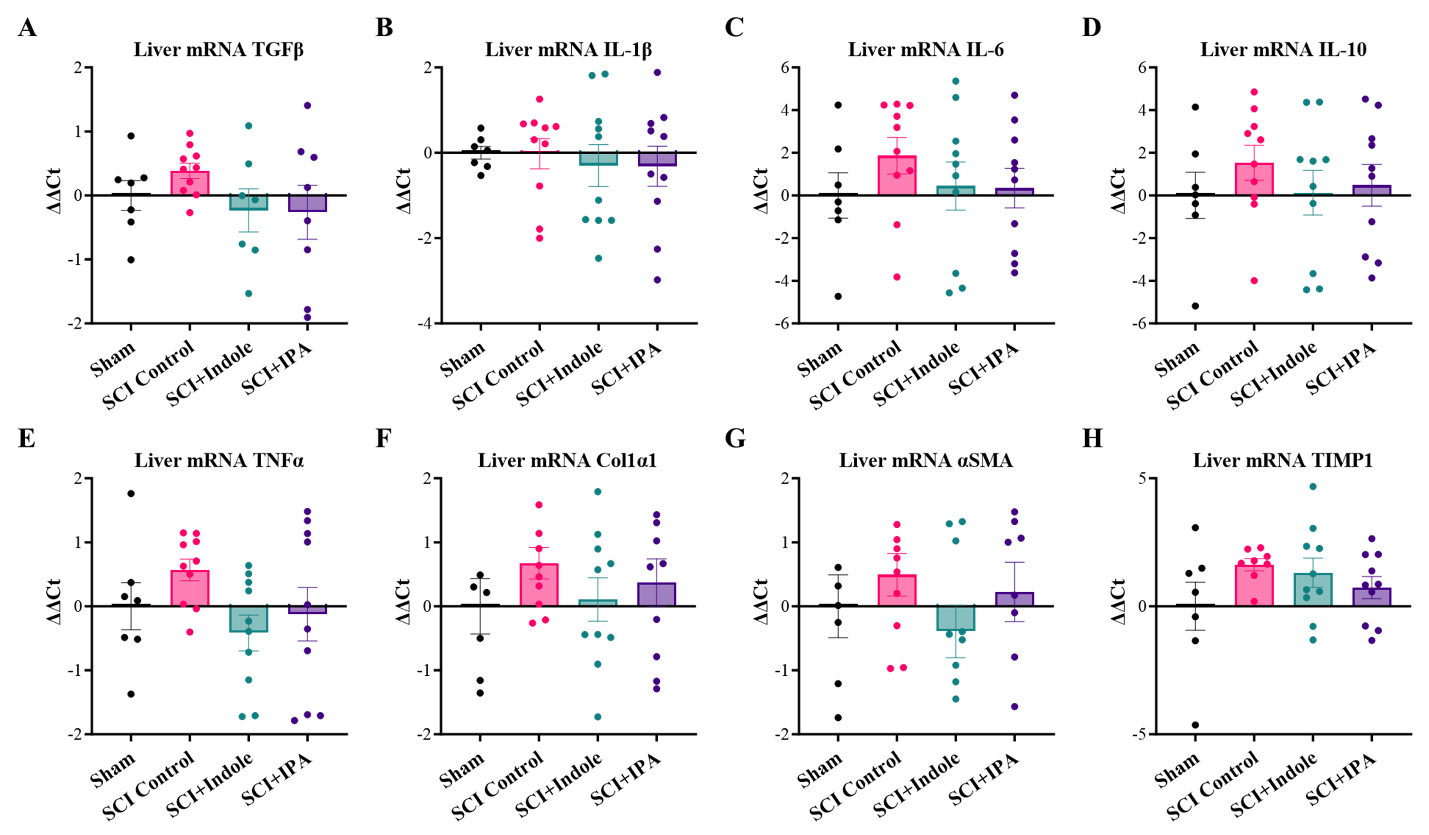
 Supplementary Figure 3. Real-time quantitative polymerase chain reaction (RT-qPCR) from livers at 6-weeks post-SCI.** mRNA levels of (A) TGFβ, (B) IL-1β, (C) IL-6, (D) IL-10, (E) TNF-α, (F) Col1α1, (G) αSMA, (H) TIMP1. Data presented as ΔΔCt. No statistical differences in mRNA levels in SCI or treatment groups. Data presented as mean ± S.E.M.; n=6-10. Data analyzed by one-way ANOVA with Tukey’s multiple comparisons test.

**Supplementary Table 4. Pairwise PERMANOVA results of hepatic mRNA inflammatory markers.**

|  | **F.Model** | **R2** | **pval** | **p.adj** |
| --- | --- | --- | --- | --- |
| SCI Control vs SCI+Indole | 1.89890 | 0.09543 | 0.16200 | 0.61200 |
| SCI Control vs SCI+IPA | 1.47690 | 0.07583 | 0.20400 | 0.61200 |
| SCI Control vs Sham | 1.08040 | 0.06719 | 0.35700 | 0.71400 |
| SCI+Indole vs SCI+IPA | 0.11930 | 0.00658 | 0.77100 | 0.77100 |
| SCI+Indole vs Sham | 0.48135 | 0.03109 | 0.52500 | 0.77100 |
| SCI+IPA vs Sham | 0.23543 | 0.01545 | 0.74000 | 0.77100 |
