# Supplementary material for: Leveraging microbiota-metabolites to reduce inflammation and promote functional recovery following spinal cord injury in female mice
Source: Brain Behav Immun Health. 2025 Dec 2;50:101157. doi: 10.1016/j.bbih.2025.101157 (PMC12719675; doi:10.1016/j.bbih.2025.101157)
Supplement: Multimedia component 2 [file mmc2.docx]

**Supplemental Table S5. Two-way ANOVA multiple comparison p-values for 7 dpi spinal cord CD68+ Area (%).**

| **Distance from Injury Site (mm)** | **-1.6** | **-1.4** | **-1.2** | **-1.0** | **-0.8** | **-0.6** | **-0.4** | **-0.2** | **0.2** | **0.4** | **0.6** | **0.8** | **1.0** | **1.2** | **1.4** | **1.6** |
| --- | --- | --- | --- | --- | --- | --- | --- | --- | --- | --- | --- | --- | --- | --- | --- | --- |
| Sham v SCI Control | 0.8803 | 0.7305 | 0.2969 | 0.0008 | <0.0001 | <0.0001 | <0.0001 | <0.0001 | <0.0001 | <0.0001 | <0.0001 | <0.0001 | <0.0001 | 0.0003 | 0.0082 | 0.0522 |
| Sham v SCI+Indole | 0.9578 | 0.7909 | 0.4658 | 0.0962 | 0.0286 | 0.0012 | <0.0001 | <0.0001 | <0.0001 | 0.0023 | 0.0291 | 0.0944 | 0.4409 | 0.6236 | 0.5977 | 0.8872 |
| Sham v SCI+IPA | 0.9923 | 0.9811 | 0.8666 | 0.2433 | 0.0337 | 0.003 | 0.0002 | 0.0003 | 0.0007 | <0.0001 | 0.0013 | 0.0227 | 0.1143 | 0.2958 | 0.5937 | 0.9055 |
| SCI Control v SCI+Indole | 0.9954 | 0.9996 | 0.9906 | 0.4101 | 0.0022 | <0.0001 | <0.0001 | 0.005 | 0.041 | <0.0001 | <0.0001 | <0.0001 | 0.0002 | 0.0183 | 0.206 | 0.255 |
| SCI Control v SCI+IPA | 0.9604 | 0.8981 | 0.7133 | 0.1345 | 0.0006 | <0.0001 | <0.0001 | <0.0001 | <0.0001 | <0.0001 | <0.0001 | <0.0001 | 0.0015 | 0.0511 | 0.157 | 0.1885 |
| SCI+Indole v SCI+IPA | 0.9947 | 0.9363 | 0.8777 | 0.9459 | 0.9978 | 0.9758 | 0.9022 | 0.3108 | 0.1391 | 0.7732 | 0.8368 | 0.9712 | 0.9046 | 0.9592 | >0.9999 | 0.9998 |

**Supplemental Table S6. Two-way ANOVA multiple comparison p-values for 42 dpi CD68 Proportional Area (%).**

| **Distance from Injury Site (mm)** | **-1.6** | **-1.4** | **-1.2** | **-1.0** | **-0.8** | **-0.6** | **-0.4** | **-0.2** | **0.2** | **0.4** | **0.6** | **0.8** | **1.0** | **1.2** | **1.4** | **1.6** |
| --- | --- | --- | --- | --- | --- | --- | --- | --- | --- | --- | --- | --- | --- | --- | --- | --- |
| Sham v SCI Control | 0.1475 | 0.0422 | 0.0078 | <0.0001 | <0.0001 | <0.0001 | <0.0001 | <0.0001 | <0.0001 | <0.0001 | <0.0001 | <0.0001 | 0.0003 | 0.0497 | 0.3072 | 0.5316 |
| Sham v SCI+Indole | 0.0897 | 0.0363 | 0.003 | <0.0001 | <0.0001 | <0.0001 | <0.0001 | <0.0001 | <0.0001 | <0.0001 | <0.0001 | <0.0001 | 0.0052 | 0.0666 | 0.309 | 0.4187 |
| Sham v SCI+IPA | 0.5139 | 0.3446 | 0.0697 | 0.0028 | <0.0001 | <0.0001 | <0.0001 | <0.0001 | <0.0001 | <0.0001 | <0.0001 | 0.0038 | 0.1685 | 0.5901 | 0.8464 | 0.8829 |
| SCI Control v SCI+Indole | 0.9977 | >0.9999 | 0.9962 | 0.9594 | 0.6748 | 0.8084 | 0.1708 | 0.0015 | 0.0154 | 0.1406 | 0.3268 | 0.8654 | 0.8138 | 0.9978 | >0.9999 | 0.9984 |
| SCI Control v SCI+IPA | 0.8306 | 0.6812 | 0.8096 | 0.4579 | 0.3044 | 0.4499 | 0.0455 | 0.0018 | 0.0529 | 0.0052 | 0.0028 | 0.0715 | 0.1133 | 0.4504 | 0.7347 | 0.9002 |
| SCI+Indole v SCI+IPA | 0.7113 | 0.6654 | 0.6632 | 0.7518 | 0.9217 | 0.9309 | 0.9389 | >0.9999 | 0.9675 | 0.6183 | 0.246 | 0.321 | 0.5019 | 0.5436 | 0.7458 | 0.8159 |

**Supplemental Table S7. Two-way ANOVA multiple comparison p-values for GFAP Mean Intensity.**

| **Distance from Injury Site (mm)** | **-1.6** | **-1.4** | **-1.2** | **-1.0** | **-0.8** | **-0.6** | **-0.4** | **-0.2** | **0.2** | **0.4** | **0.6** | **0.8** | **1.0** | **1.2** | **1.4** | **1.6** |
| --- | --- | --- | --- | --- | --- | --- | --- | --- | --- | --- | --- | --- | --- | --- | --- | --- |
| Sham v SCI Control | >0.9999 | 0.9995 | 0.9987 | 0.9216 | 0.606 | 0.3263 | 0.0053 | 0.0038 | 0.0161 | 0.0255 | 0.3433 | 0.8361 | 0.9537 | >0.9999 | 0.9993 | 0.9973 |
| Sham v SCI+Indole | >0.9999 | 0.99 | 0.9715 | 0.9669 | 0.8714 | 0.7513 | 0.257 | 0.0251 | 0.0111 | 0.1602 | 0.8745 | 0.9961 | 0.8678 | 0.7004 | 0.6532 | 0.8459 |
| Sham v SCI+IPA | >0.9999 | 0.9919 | 0.9718 | 0.8004 | 0.5468 | 0.2173 | 0.0048 | 0.002 | 0.0099 | 0.0472 | 0.8173 | 0.9986 | 0.9971 | 0.7472 | 0.7397 | 0.7574 |
| SCI Control v SCI+Indole | >0.9999 | 0.9969 | 0.9195 | 0.9973 | 0.9505 | 0.858 | 0.3389 | 0.8923 | >0.9999 | 0.825 | 0.7399 | 0.6534 | 0.5003 | 0.6822 | 0.6843 | 0.9116 |
| SCI Control v SCI+IPA | >0.9999 | 0.9978 | 0.92 | 0.9923 | 0.9999 | 0.9968 | >0.9999 | 0.9995 | 0.9997 | 0.99 | 0.8117 | 0.8766 | 0.8597 | 0.7333 | 0.7741 | 0.8347 |
| SCI+Indole v SCI+IPA | >0.9999 | >0.9999 | >0.9999 | 0.9614 | 0.9253 | 0.7338 | 0.3413 | 0.8313 | >0.9999 | 0.9412 | 0.9992 | 0.9752 | 0.9232 | 0.9998 | 0.9986 | 0.9977 |

**Supplemental Table S8. Two-way ANOVA multiple comparison p-values for Sox9+ Cells/mm^2.**

| **Distance from Injury Site (mm)** | **-1.6** | **-1.4** | **-1.2** | **-1.0** | **-0.8** | **-0.6** | **-0.4** | **-0.2** | **0.2** | **0.4** | **0.6** | **0.8** | **1.0** | **1.2** | **1.4** | **1.6** |
| --- | --- | --- | --- | --- | --- | --- | --- | --- | --- | --- | --- | --- | --- | --- | --- | --- |
| Sham v SCI Control | 0.0233 | 0.0422 | 0.0045 | 0.0002 | <0.0001 | <0.0001 | <0.0001 | 0.022 | 0.0018 | <0.0001 | <0.0001 | 0.0005 | 0.0075 | 0.0243 | 0.0672 | 0.1514 |
| Sham v SCI+Indole | 0.1951 | 0.0487 | 0.0046 | 0.0005 | <0.0001 | <0.0001 | <0.0001 | 0.0013 | <0.0001 | <0.0001 | <0.0001 | 0.0011 | 0.0088 | 0.0239 | 0.128 | 0.0562 |
| Sham v SCI+IPA | 0.161 | 0.1082 | 0.0261 | 0.0033 | <0.0001 | <0.0001 | <0.0001 | <0.0001 | <0.0001 | <0.0001 | <0.0001 | 0.0056 | 0.0145 | 0.0642 | 0.131 | 0.1022 |
| SCI Control v SCI+Indole | 0.7897 | >0.9999 | >0.9999 | 0.9915 | 0.9817 | 0.776 | 0.9943 | 0.8025 | 0.6282 | 0.9065 | 0.9932 | 0.9966 | >0.9999 | >0.9999 | 0.9912 | 0.9691 |
| SCI Control v SCI+IPA | 0.8059 | 0.9656 | 0.9046 | 0.7788 | 0.9982 | 0.9928 | 0.4807 | 0.1132 | 0.2069 | 0.5291 | 0.8553 | 0.8606 | 0.9904 | 0.9692 | 0.9833 | 0.999 |
| SCI+Indole v SCI+IPA | >0.9999 | 0.9765 | 0.9078 | 0.9155 | 0.9443 | 0.8957 | 0.3296 | 0.5535 | 0.887 | 0.1725 | 0.9538 | 0.9415 | 0.9948 | 0.9679 | >0.9999 | 0.9882 |

**Supplemental Table S9. Two-way ANOVA multiple comparison p-values for NeuN+ Cells/mm^2.**

| **Distance from Injury Site (mm)** | **-1.6** | **-1.4** | **-1.2** | **-1.0** | **-0.8** | **-0.6** | **-0.4** | **-0.2** | **0.2** | **0.4** | **0.6** | **0.8** | **1.0** | **1.2** | **1.4** | **1.6** |
| --- | --- | --- | --- | --- | --- | --- | --- | --- | --- | --- | --- | --- | --- | --- | --- | --- |
| Sham v SCI Control | 0.7475 | 0.5408 | 0.2444 | 0.1913 | 0.0038 | <0.0001 | <0.0001 | <0.0001 | <0.0001 | <0.0001 | <0.0001 | <0.0001 | <0.0001 | <0.0001 | <0.0001 | <0.0001 |
| Sham v SCI+Indole | 0.7248 | 0.5839 | 0.2765 | 0.3345 | 0.0538 | <0.0001 | <0.0001 | <0.0001 | <0.0001 | <0.0001 | <0.0001 | <0.0001 | 0.0009 | 0.0083 | 0.011 | 0.0254 |
| Sham v SCI+IPA | 0.9638 | 0.6695 | 0.394 | 0.2718 | 0.0364 | 0.0017 | <0.0001 | <0.0001 | <0.0001 | <0.0001 | <0.0001 | <0.0001 | 0.0002 | 0.0023 | 0.0109 | 0.0031 |
| SCI Control v SCI+Indole | >0.9999 | 0.9998 | 0.9998 | 0.9852 | 0.7675 | 0.6198 | 0.6281 | 0.9999 | >0.9999 | 0.6112 | 0.2411 | 0.1209 | 0.2364 | 0.1342 | 0.3271 | 0.1234 |
| SCI Control v SCI+IPA | 0.9278 | 0.9939 | 0.9827 | 0.9939 | 0.8109 | 0.1251 | 0.4578 | >0.9999 | >0.9999 | 0.5124 | 0.3302 | 0.2047 | 0.4247 | 0.2371 | 0.2709 | 0.3819 |
| SCI+Indole v SCI+IPA | 0.9129 | 0.9979 | 0.9915 | 0.9996 | 0.9996 | 0.7747 | 0.9952 | 0.9994 | >0.9999 | 0.9993 | 0.9956 | 0.9895 | 0.9767 | 0.9864 | 0.9999 | 0.9098 |

**Supplemental Table S10. Two-way ANOVA multiple comparison p-values for 5HT Mean Intensity.**

| **Distance from Injury Site (mm)** | **-1.6** | **-1.4** | **-1.2** | **-1.0** | **-0.8** | **-0.6** | **-0.4** | **-0.2** | **0.2** | **0.4** | **0.6** | **0.8** | **1.0** | **1.2** | **1.4** | **1.6** |
| --- | --- | --- | --- | --- | --- | --- | --- | --- | --- | --- | --- | --- | --- | --- | --- | --- |
| Sham v SCI Control | >0.9999 | 0.9967 | 0.9656 | 0.9974 | 0.9646 | 0.7122 | 0.999 | 0.0081 | 0.0004 | 0.0003 | 0.0006 | 0.0005 | 0.0023 | 0.0132 | 0.0266 | 0.0213 |
| Sham v SCI+Indole | >0.9999 | 0.7756 | 0.9325 | 0.9767 | 0.839 | 0.7327 | >0.9999 | 0.0491 | 0.0004 | 0.0002 | 0.0007 | 0.0036 | 0.0293 | 0.165 | 0.2319 | 0.1303 |
| Sham v SCI+IPA | >0.9999 | 0.9955 | 0.9995 | 0.948 | 0.9995 | 0.9981 | >0.9999 | 0.0012 | <0.0001 | 0.0005 | 0.002 | 0.0071 | 0.0442 | 0.0827 | 0.1315 | 0.2222 |
| SCI Control v SCI+Indole | >0.9999 | 0.8537 | 0.9993 | 0.9958 | 0.982 | 0.9999 | 0.9985 | 0.877 | 0.9998 | >0.9999 | 0.9987 | 0.9143 | 0.7768 | 0.6659 | 0.7104 | 0.8346 |
| SCI Control v SCI+IPA | >0.9999 | >0.9999 | 0.9118 | 0.8481 | 0.9122 | 0.7377 | 0.9991 | 0.9752 | 0.8379 | 0.9894 | 0.9492 | 0.7696 | 0.6271 | 0.8177 | 0.8412 | 0.6288 |
| SCI+Indole v SCI+IPA | >0.9999 | 0.8392 | 0.8469 | 0.7006 | 0.7034 | 0.7588 | >0.9999 | 0.6113 | 0.7758 | 0.9834 | 0.9791 | 0.9902 | 0.9956 | 0.9906 | 0.9929 | 0.9857 |
